# Assessing the technical efficiency performance of Chinese ports logistics: Evidence from the DEA and fsQCA

**DOI:** 10.1371/journal.pone.0300655

**Published:** 2024-04-16

**Authors:** Yi Xie, Ren Hu

**Affiliations:** 1 Department of Finance, Faculty of Business, Suzhou Institute of Technology, Jiangsu University of Science and Technology, Jiangsu, China; 2 Department of Logistics Management, Faculty of Business, Jiangsu University of Science and Technology, Jiangsu, China; Massey University, NEW ZEALAND

## Abstract

Ports are critical centers of international trade and global logistics now that economic globalization has taken hold. The efficiency performance of port logistics is crucial to building an emerging pattern of development in which domestic and foreign dual cycles are complementary for China. This paper examines the efficiency performance of 19 ports within five major economic circles in China. It explores how their efficiency is distributed, and the configurations of efficiency improvement during the new normal of China’s economy. First, the DEA-BCC model is employed to calculate the technical efficiency performance and distribution of each port from 2011 to 2020. Then, fuzzy-set qualitative comparative analysis (fsQCA) was applied to integrate and analyze the influencing factors. The results show as follows: (1) Each port group performed differently on efficiency rankings, as well as regional distributions. Among these, the port groups of the Bohai Rim region, the Yangtze River Delta region, and the Bohai Rim region continue to rank highly. (2) From the perspective of configuration analysis, the results suggest that government support is not necessary for port logistics with better economic endowments. However, it is critical for backward ones. (3) A rational industrial structure can enhance levels of infrastructure, openness, and information technology, improving port performance. The findings can provide theoretical and practical references for better promoting the development of Chinese port management.al references for better promoting the development of Chinese port management.

## 1 Introduction

Ports, as the meeting points of maritime logistics and the conversion nodes of passenger and cargo waterway transportation, play a significant role in the national transportation system. In addition, they play a significant role in the national economy’s development. Statistics from China’s transportation department indicate that port container throughput in 2021 reached 0.264 billion TEUs, an increase of 1.2% year-on-year, and port cargo throughput and container throughput ranked first in the world. Although China has become a driving force for the development of global logistics, and the cost of social logistics shows a trend of declining, there is still space for improvement compared with developed countries. According to the Ministry of Transportation in China, over 95% of China’s foreign trade was completed through port transportation in 2021. Taking the new normal of the economy into account, the development speed of the Chinese economy is slowing down [1, 2], or in other words, industrial development should likewise adjust to changed market conditions. As a basic industry supporting the development of the national economy [3, 4], the logistics industry promotes the further improvement of the efficiency of the logistics industry and realizes the transformation and upgrading of the logistics industry. Therefore, it is of practical significance to evaluate and compare the efficient distribution of port logistics in different regions, figure out the improvement path of high-level efficiency of the port logistics industry, and identify the key factors affecting the efficiency performance of the logistics industry, to promote the high-quality development of port logistics in China.

Previous research on port logistics efficiency mainly focused on the hard environment of ports, including infrastructure [5], port supply chain [6], the spatial and temporal structural characteristics of port logistics efficiency [7], and some further extended to the soft environment of ports, including the interaction between port logistics and hinterland economy [8], the relationship between port logistics and regional economy [9], while the research on the combination of soft and hard environments is still limited. Considering the method used in the estimation of the efficiency performance of port logistics, since the DEA (data envelopment analysis) model to evaluate the efficiency performance of port logistics [10], it is widely employed in the measurement of efficiency in various fields. It is found that DEA is applicable to the confidence intervals for efficiency scores [11]. The research also examined logistics efficiency with the Four-Stage Bootstrap DEA model [12]. The DEA model is also combined with the Analytic Hierarchy Process (AHP) to quantify the efficiency performance of the logistics industry in European countries [13]. The determinants of efficiency performance are regressed with the Tobit regression model in China’s logistics industry and it found that there is a positive relationship between economic and logistics development and logistics efficiency [14]. In previous academic research using traditional regression analyses, the focus has been primarily on exploring the “Net-Effects” of a single factor. Therefore, this paper examines how their efficiency is distributed and what efficiency improvements were incorporated behind port logistics development in China.

To address the gaps in prior studies, this study contributes as follows: first, most previous studies looked at port logistics in the context of hard environment factors as a factor in influencing port logistics which fills the theoretical gap in existing research. Second, in line with the prior study [15], this research expands the research framework of port logistics by constructing a more systematic framework for analyzing the contributing factors of both the hard environment and soft environment in port logistics and provides practical implications regarding the distribution of port logistics efficiency in five economic circles. Finally, prior studies on logistics focused on the “Net-Effect” of a single influencing factor on port logistics efficiency performance. In addition to the common DEA-BCC method, the fsQCA method is applied in this study to find the applicable path to improve port logistics efficiency in China. Since fsQCA has advantages in the subtle effects of various conditions [16], the finding provides solutions and ideas for how can China’s ports logistics upgrade the efficiency performance.

The rest of this research is arranged as follows: The review of relevant literature is presented in Section 2, followed by data and research design in Section 3, and the empirical analysis of port logistics efficiency is demonstrated in Section 4. Section 5 identifies the improvement path for port logistics efficiency. Finally, the conclusion and discussion are discussed in Section 6.

## 2 Literature review

Port logistics is the logistics provided by the port with advanced software and hardware environments [17], strengthening its radiation ability to logistics activities around the port, based on the surrounding industries of the port, forming a comprehensive port service system covering all aspects of the chain of port enterprises, and using information technology to achieve the purpose of optimizing and integrating port resources. With the development of economic globalization, logistics improvement plays a critical role in promoting trade between countries and regions. Logistics efficiency research has attracted much attention but is mostly focused on one region without considering the distribution of closely connected regions. The efficiency of the logistics industry in Anhui Province with the DEA method and then the Tobit regression method is adopted to reveal the influencing factors [18]. The efficiency of logistics for high-quality development in Jiangxi province using a three-stage DEA method [19]. The Korean ports’ logistics with is examined with configuration analysis [20]. It can be found that the former research on the logistics industry efficiency measurement is mature, considering the positive interaction between cities and ports in the economic circle [21]. However, the research on the logistics efficiency of port clusters in the economic circle is still limited in this study.

It is conclued that the claim is more critical for logistics efficiency improvement in remote areas [22]. The improvement of logistics efficiency in remote areas should focus on infrastructure development and the effective use of resources. The development of infrastructure requires increased investment from the government. The DEA-Malmquist combined with fsQCA is employed to measure logistics efficiency in China from both inside and outside the industry [23]. The location advantage of industrial agglomerations impacts pure technical efficiency, while the volume of economic activity is related to scale efficiency [24]. It is noted that the external environmental factors have a more significant positive impact on port efficiency in the Yangtze River Delta region than internal ones [25]. In this regard, the current literature focuses on the effect of a single influencing factor. However, the grouping effect between various influencing factors on logistics efficiency is not fully investigated.

Overall, research on logistics efficiency measurement is about a single area, and its influencing factors are mainly focused on the “Net-Effects” of a single factor, while fsQCA can find the configuration relationship between a variety of factors [26]. Therefore, this research employs the DEA-BCC model to evaluate port logistics efficiency performance and analyze the distribution of port logistics efficiency in five port clusters in China. This research focuses on 19 ports with 5 economic circles as the research objective. Afterward, a framework with both hard and soft environments is developed for port logistics enterprises. Furthermore, the efficiency evaluation results are considered explanatory variables, and fsQCA is employed to explore the underlying factors and mechanisms and construct specific solutions to improve port logistics efficiency in China.

## 3 Research design

### 3.1 DEA-BCC

Data envelopment analysis (DEA) is a nonparametric frontier research method, which is considered as a measure of comparative efficiency or composite benchmark when multiple performance indicators exist in a decision-making unit (DMU) [27]. Since port policies are still being refined, there is imperfect competition in the market, and ports operate under non-ideal conditions, it is impossible to determine whether the port is in a situation that is increasing or decreasing returns to scale. Therefore, considering the various internal structures, the DEA-BBC model proposed by Banker, Cooper, and Charnes (1984) is employed to examine the technical efficiency (CRSTE), pure technical efficiency (VRSTE) and scale efficiency (SCALE) of port logistics in this study. The DEA-BBC model of the port logistics industry is expressed as below.


$$\min Z_{a}=\delta_{a}-\rho \sum_{i=1}^{k}p_{ia}^{-}+\rho \sum_{i=1}^{k}p_{ra}^{-}$$ (1)

$$p,q.\{\begin{array}{l} \sum_{j-1}^{n}x_{ij}\gamma_{j}p_{i}^{-}=\delta x_{i0}\\ \sum_{j-1}^{n}y_{rj}\gamma_{j}-p_{r}^{+}=\delta y_{r0}\\ \sum_{j-1}^{n}=1\\ \gamma_{j},p_{i}^{-},p_{r}^{+}⩾0\\ j=1,2,\cdots ,n;i=1,2,\cdots ,m;r=1,2,\cdots ,s \end{array}$$ (2)

Where j is taken as the number of each decision unit in each port, where each port has i categories of inputs and r categories of outputs. Thus, the a-dimensional vector X_ij_ and the b-dimensional vector Y_rj_ represent the input and output of the jth port, respectively. Based on the relative efficiency Z_a_ in Eq (1), the slack variables p+ and p- for each input, the efficiency evaluation index δ, and the non-Archimedean infinitesimal value p are introduced. and the DMU is valid if Z_a_ = 1; if Z_a_ < 1, the DMU is in an invalid state.

#### 3.1.1 Data

Sample data for this study were collected from the Yearbook of China’s Ports, the Yearbook of China’s Energy, and the National Economic and Social Development Statistical Bulletin in the period of 2010–2020, covering five economic circles in China, namely the Bohai Sea Rim (Dalian Port, Yingkou Port, Tianjin Port, Yantai Port, Qingdao Port), the Southeast Coast (Xiamen Port, Fuzhou Port, Quanzhou Port), the Yangtze River Delta (Shanghai Port, Ningbo Port, Suzhou Port, Nanjing Port, Lianyungang Port), the Pearl River Delta (Guangzhou Port, Shenzhen Port, Zhuhai Port), and the Southwest Coast (Zhanjiang Port, Sanya Port, Haikou Port), which are corresponding to five port clusters. Finally, the final sample selection led to panel data from 19 ports, with 209 observations on efficiency measurements, namely operational efficiency (CRS), pure technical efficiency (VRS), and scale efficiency (SCALE).

#### 3.1.2 Variables selection: Inputs and outputs

To include the most appropriate items of the port logistics system for efficiency measurements, we regard the inputs of the DEA-BCC model as: i) Port throughput capacity (x_1_) [15]; ii) Number of production berths (x_2_) [28]; and iii) Infrastructure investment (x_3_) [15]. While the outputs of the port logistics system are as follows: i) Cargo throughput (y_1_) [28]; ii) Container throughput (y_2_) [29]; iii) Port cargo turnover (y_3_) [15]; and ⅳ) Port cargo volume (y_4_) [30]. The descriptive statistics of the inputs and outputs are shown in Table 1.

**Table 1:** Descriptive statistics of inputs and outputs.

Variables		Obs.	Mean	S.D.	Max	Min
Inputs	Port throughput capacity	209	338.79	251.24	1001.66	1.54
	No. of production berth	209	193.68	172.71	627.00	3.00
	Infrastructure investment	209	4.74	3.29	14.10	0.51
Outputs	Cargo throughput	209	85.25	95.93	435.03	0.77
	Container throughput	209	249.96	185.82	831.97	1.28
	Port cargo turnover	209	218.55	494.18	3209.46	9.59
	Port cargo volume	209	102.95	132.71	734.79	0.11

### 3.2 Fuzz-set QCA

Qualitative comparative analysis (QCA) allows complex causal relationships to be explored by small sample scores, emphasizing empirical and theoretical correlations to solve relevant problems [31]. The technique contains four specific methods, namely clear set qualitative comparative analysis (csQCA), multi-value set qualitative comparative analysis (mvQCA), fuzzy set qualitative comparative analysis (fsQCA), and MSDO/MDSO (maximum similarity, different outcome, maximum difference, same outcome). In this paper, fsQCA is used to make analysis results more representative through the necessity detection function of univariates in QCA. Consistency and Coverage are the two main detection indicators of the fsQCA model. Consistency means that all cases involved in the comparative analysis can be jointly explained by a single condition that underlies the results. The consistency of the necessary fuzzy subset analysis lies in the existence of sufficient or necessary relationships. Coverage refers to the reliability of a single conditional variable to explain the occurrence of the outcome variable. The consistency test for the necessary fuzzy subset relationship is shown below.


$$Consistency(Y_{i}\leq X_{i})=\sum \left(\min(X_{i},Y_{i})\right)/\sum \left(Y_{i}\right)$$ (3)

Where Y_i_ denotes the subordination of situation i to the outcome variable and X_i_ denotes the subordination of situation i to the precondition. In this paper, the efficiency values of five economic circles corresponding to five port clusters, totaling 19 ports, derived from the above model are used as outcome variables. The condition variables are divided according to hard environment and soft environment, which are measured by applying the fsQCA model. The final data were obtained from the China Port Yearbook, China Marine Statistical Yearbook, and the statistical bulletins of each port city in 2020.

#### 3.2.1 Variables selection

Considering the outcome variable, technical efficiency is a comprehensive indicator that includes both pure technical efficiency and scale efficiency. This reflects the port logistics industry’s growth conditions. The outcome variable in this research is the technical efficiency score derived from the DEA technique. This consists of 19 ports in 2020. As for the outcome variables, since too many conditions and variables will lead to the generation of combined samples exceeding the total conditions and not reflecting the real situation, this paper considers the integrity and continuity of the data based on the analysis of the factors influencing logistics efficiency in the literature. In this paper, we adopt the study of port logistics based on Li et al. (2021) and divide the influencing factors of port logistics efficiency into two segments, including hard environment and soft environment.

**Fig 1:**
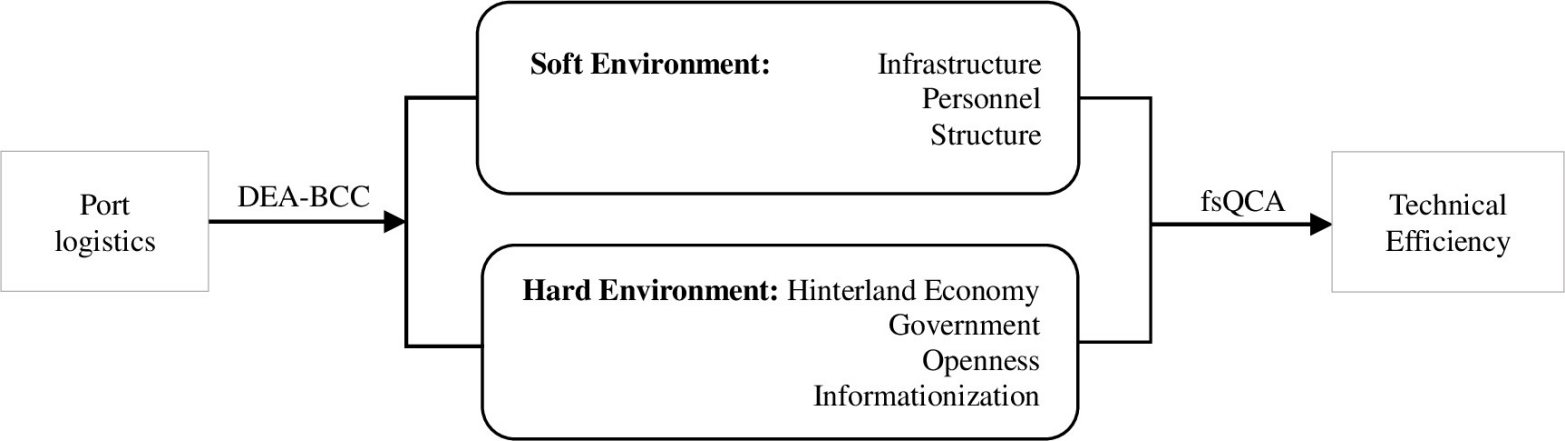
Determinants for the efficiency of port logistics.

The conditional variables within a hard environment are: i) Infrastructure [28], which consists of entropy-weighted composite value by the number of berths for production, the number of 10,000-ton berths, the length of production quays, and the water depth of waterways; ii) Personnel [32], which corresponds to the volume of water transport enterprises in ports; iii) Structure [23], which refers to the ratio of the added value of the tertiary industry to the GDP of each region. The variables of the hard environment include: i) Hinterland Economy [28], which is explained by the GDP per capita of the region; ii) Government [28], which includes budget invested by the government in the port where the port is located; iii) Openness [20], which refers to the import and export volume of the city where the port located; ⅳ) Informationization [28], it is explained by the number of Internet broadband access ports is used to illustrate the digital transformation. Since the conditional variables in this paper are continuous variables, fsQCA can more fully capture the subtle effects brought by the changes of antecedent conditions at different levels or degrees [16]. Fig 1 illustrates the research process of this study.

## 4 Empirical analysis

This paper examines the efficiency performance of China’s port logistics and explore how their efficiency distributed in five regions. The summary of technical efficiency (CRSTE), pure technical efficiency (VRSTE), and scale efficiency (SCALE) is presented in Table 2.

**Table 2:** Distribution of port clusters by efficiencies.

Port Group	City	CRS	Rank	VRS	Rank	SCALE	Rank
Bohai Sea Rim	Dalian	0.992	8	0.997	9	0.994	9
	Yingkou	1.000	1	1.000	1	1.000	1
	Tianjin	0.995	7	1.000	7	0.995	8
	Yantai	0.899	15	0.911	17	0.986	13
	Qingdao	0.973	10	0.975	12	0.998	6
Southeast Coast	Xiamen	0.895	17	0.925	16	0.970	15
	Fuzhou	0.927	14	0.937	15	0.989	12
	Quanzhou	1.000	1	1.000	1	1.000	1
Yangtze River Delta	Shanghai	1.000	1	1.000	1	1.000	1
	Ningbo	0.966	11	0.993	11	0.973	14
	Suzhou	1.000	1	1.000	1	1.000	1
	Nanjing	0.756	20	0.854	18	0.894	19
	Lianyungang	0.898	16	0.969	14	0.927	17
Pearl River Delta	Guangzhou	0.811	19	0.818	19	0.991	11
	Shenzhen	1.000	1	1.000	1	1.000	1
	Zhuhai	0.936	13	0.994	10	0.941	16
Southwest Coast	Zhanjiang	0.893	18	0.971	13	0.920	18
	Sanya	0.996	6	1.000	6	0.996	7
	Haikou	0.991	9	0.998	8	0.993	10
Average		0.944		0.965		0.977	

### 4.1 Operating efficiency

**Fig 2:**
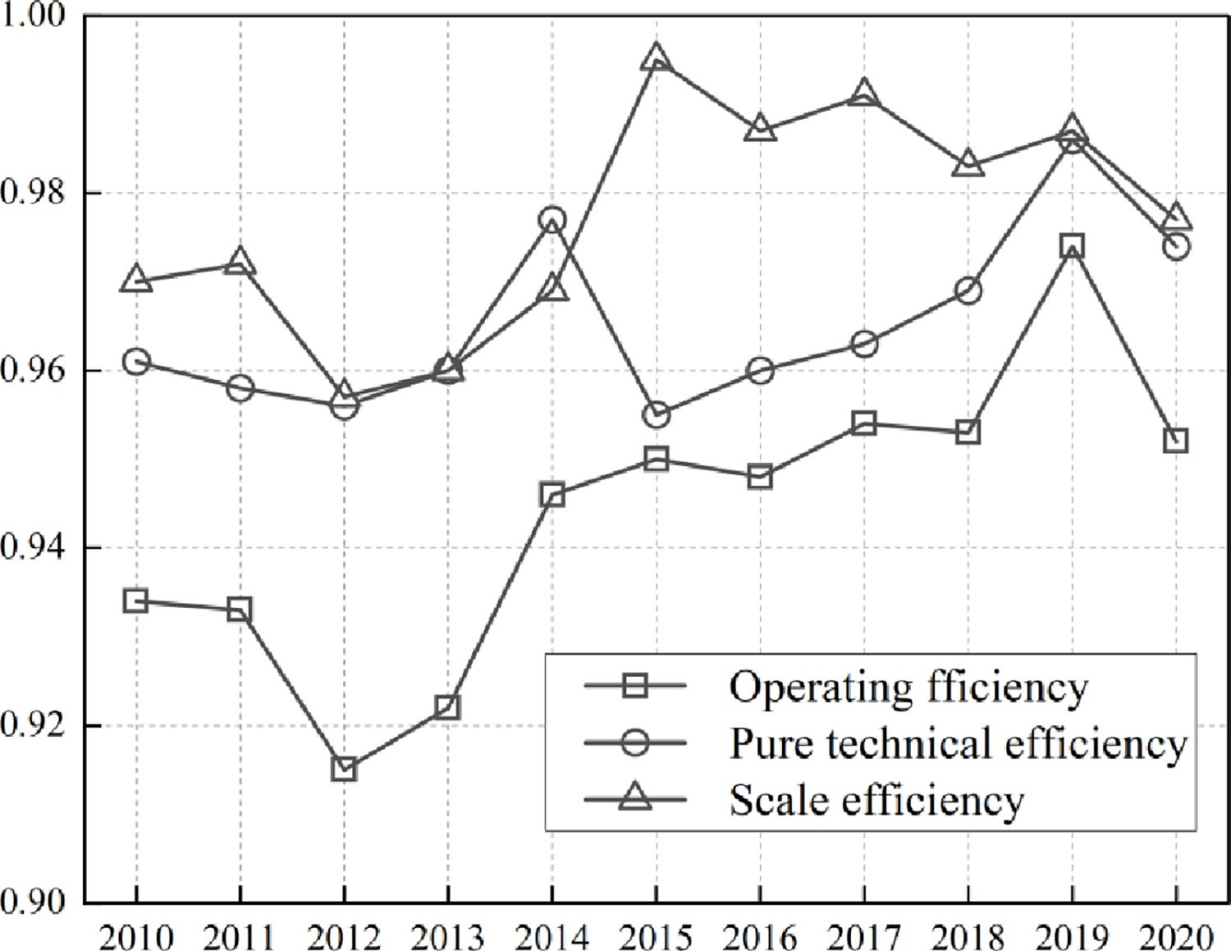
Distribution of efficiency scores from 2010 to 2020.

Technical efficiency (CRSTE) is an influential indicator of port logistics, as it reflects the ability to allocate and utilize resources effectively. Fig 2 shows that the average score of CRSTE from 2010 to 2020 is 0.944 and shows fluctuating growth. This indicates that the resource allocation efficiency of each region is high and that the input-output structures are still being strengthened. The results in Table 2 demonstrate that the average value of technical efficiency is arranged as Bohai Rim > Southwest Coast > Southeast Coast > Yangtze River Delta > Pearl River Delta. Of these, Bohai Rim and Southwest Coast outperform other regions with the mean value of CRSTE at 0.944. This means that they used reasonable resources and had reasonable management. Meanwhile, the efficiency score of the Southeast Coastal region lags on the effective frontier and is below the average value of the total sample. It indicates that CRSTE is unevenly developed in China’s port logistics and competition is fierce in the market. With the implementation of “Guiding Opinions on Deepening Pan-Pearl River Delta Regional Cooperation” issued by the State Council in 2016, government support enhanced resource allocation and competitiveness within this economic circle. The port cities of Yingkou, Quanzhou, Shanghai, Suzhou, and Shenzhen maintain a technical efficiency of one. This indicates that these port cities have optimally utilized their logistics inputs. The five last cities are Lianyungang, Xiamen, Zhanjiang, Guangzhou, and Nanjing, showing a significant impact on the technical efficiency of these cities because of their input-output structures. There is surplus input and waste of resources, still room for further optimization.

### 4.2 Pure technical efficiency (VRSTE)

CRSTE was further decomposed into pure technical efficiency (VRSTE) and scale efficiency (SCALE) under the assumption of constant returns to scale [33]. These concepts reflect the management, institutional, and scale levels of each region. According to Fig 2 and Table 2, the average value of pure technical efficiency of port logistics during 2010–2020 is 0.965, which is much lower than the average value of scale efficiency. Additionally, it shows a fluctuating upward trend. This indicates that each port group’s scale is relatively high, but pure technical efficiency is relatively low. The overall pure technical efficiency fluctuates between 0.955 and 0.986, indicating that the level of pure technical efficiency is relatively stable. In terms of pure technical efficiency among port clusters, Southwest Coastal > Bohai Rim > Yangtze River Delta > Southeast Coastal > Pearl River Delta. Among them, Southwest Coast Group and Bohai Rim Group have higher efficiency than 0.965. Evidence indicates that the management capability of port logistics in these regions outperforms others, by utilizing a more rational use of inputs and maximizing output. The Yangtze River Delta port group performs slightly worse than the average. The implementation of the “Yangtze River Delta Regional Integrated Development Plan Outline” in 2019 further promoted the resource allocation in this region. In contrast, the Southeast Coast and the Pearl River Delta port groups perform more poorly in terms of pure technical efficiency of port logistics in relative terms. Table 2 shows seven ports that maintain a technical efficiency of one. They are Yingkou, Quanzhou, Shanghai, Suzhou, Shenzhen, Sanya, and Tianjin. In contrast, the last five ports Fuzhou, Xiamen, Yantai, Nanjing, and Guangzhou, make the most disrespectful effort on input-output turnovers to upgrade their management capability among these sample cities. This worsens their performance in technical efficiency. There is surplus input and severe resource waste, but there is still room for further optimization.

### 4.3 Scale efficiency (SCALE)

Scale efficiency (SCALE) represents the distance between the production frontier with changes in return to scale and the production frontier where the return to scale is constant. It can be observed from Fig 2 that the scale efficiency fluctuates between 0.957 and 0.995. This indicates that Chinese port logistics optimization is faster. In addition, changes in scale efficiency are more consistent with changes in operational efficiency in the process of efficiency change. This indicates that scale efficiency optimization is the most influential factor in improving operating efficiency and port logistics. Additionally, Table 2 illustrates that five ports have attained a scale efficiency of one. They are Yingkou, Quanzhou, Shanghai, Suzhou, and Shenzhen. Further, the scale efficiency of the Sanya and Tianjin ranges is 0.995 and 0.996, respectively. This indicates that their inefficient operation is primarily due to a poor scale level and requires further optimization. The scale efficiency scores of both Sanya and Tianjin are below 1, suggesting the inefficient operation caused by their low scale efficiency needs to be further optimized. However, the backwardness of port logistics management capability deteriorates technical efficiency and needs to be addressed. Table 2 also shows the range by Bohai Rim > Southeast Coast > Pearl River Delta > Southwest Coast > Yangtze River Delta. Furthermore, it can be observed that the Southwest Coast and the Yangtze River Delta regions have relatively low-efficiency levels and still lag behind the optimal scale. The results show that inefficient ports are more likely to be characterized by diminishing returns to scale. This implies that the production structure should be further optimized.

## 5 Fuzzy-set qualitative comparative analysis

### 5.1 Variable calibration

Since a unified standard concerning the port environment has not yet been identified, the paper employs the direct method to calibrate the variables as fuzzy sets. This is done to transform the original data between 0 and 1. Based on previous literature, this paper sets the outcome variables as the technical efficiency of port logistics and six conditional variables, namely, Infrastructure [28], Personnel [32], Structure [23], Hinterland Economy [28], Government [28], Openness [20], and Informationization [28]. In line with the former research [34], we set 0.95 as full membership, 0.5 as a crossover point, and 0.05 as full non-membership. In this study, the fuzzy sets and the frequency of acceptable cases are calibrated using the direct calibration method for the relevant conditions and outcomes. And we assign the value of foreign indicators greater than 0 as one and others equal to 0. The results of the fuzzy set calibration and descriptive statistics are shown in Table 3. The variables are calibrated into fuzzy sets by the direct method [35].

**Table 3:** Collection, calibration and descriptive analysis.

Collection	Fuzzy set calibration			Descriptive analysis			
	Full membership	Cross point	Full non- membership	Mean	S.D.	Max	Min
TE	1	0.95	0.78	0.95	0.08	1	0.78
Infrastructure	3.01	2.58	2.32	2.59	0.23	3.23	2.23
Personnel	6.67	3.63	2.365	4.25	1.66	8.34	2.32
Structure	73.88	56.07	45.47	58.43	10.74	80.50	40.60
Hinterland Economy	2877.33	1002	126.25	1159.75	1016.29	3870.06	69.54
Government	12.46	6.92	4.23	7.35	2.29	12.51	4.02
Openess	446.94	55.61	4.86	113.53	147.31	503.01	2.63
Informationization	68.93	37	7.04	34.95	22.85	89	4.70

### 5.2 Analysis of necessary conditions

In the logic minimization process, the necessary conditions must be checked before the truth table can be constructed. This enables the appropriate assumptions to be made about the logical remainder in the logic minimization process before the truth table can be constructed. According to the fsQCA model, the consistency and coverage tests determine whether there is a sufficiently high probability that there is a relationship between the conditions and the outcomes. In general, if a particular conditional variable constitutes a necessary condition for the outcome variable, the consistency score for each single condition can exceed 0.9 [36]. As shown in Table 4, the consistency values for all conditions in the high efficiency group are below 0.9. This indicates that there is no single necessary condition and is more suitable for group analysis. In contrast, there are two necessary conditions in the low-efficiency group, which are the level of economic activity in the country and the degree of Informationization. These conditions are necessary when constructing the truth table.

**Table 4:** Analysis summary of necessary conditions.

Condition	OE		~OE	
	Consistence	Coverage	Consistence	Coverage
Infrastructure	0.5484	0.8553	0.5450	0.3597
~Infrastructure	0.5894	0.7537	0.7807	0.4225
Personnel	0.6278	0.8465	0.5451	0.3111
~Personnel	0.4891	0.7176	0.7310	0.4539
Structure	0.6518	0.9034	0.4566	0.2679
~Structure	0.4718	0.6723	0.8354	0.5038
Hinterland Economy	0.6106	0.9645	0.3451	0.2307
~Hinterland Economy	0.5130	0.6493	0.9469	0.5072
Government	0.5402	0.7838	0.5945	0.3651
~Government	0.5625	0.7662	0.6480	0.3736
Openness	0.5544	0.9003	0.4619	0.3175
~Openness	0.5797	0.7180	0.8549	0.4481
Informationization	0.6128	0.9635	0.3115	0.2073
~Informationization	0.4958	0.6298	0.9451	0.5081

Note(s): “∼” indicates the absence of the condition

### 5.3 Constructing the truth table

In general, the fsQCA model generates three types of solutions, namely solutions for parsimonious, intermediate, and complex solutions [37]. These solutions differ not only in complexity, but also in revealing and generalizing. The parsimonious solutions are the most forgiving in terms of revealing solutions but are more likely to face conflicting situations [38]. Therefore, we use the intermediate solution combined with the parsimonious solution to distinguish between the core and peripheral conditions for port logistics efficiency improvement. To find the path combinations that logically satisfy the results, this paper uses fsQCA 3.0 software with the original consistency threshold of 0.8, a case frequency threshold of 1, and the proportional reduction in inconsistency (PRI) of less than 0.8. This research divides the results into high efficiency paths and non-high efficiency paths. As shown in Table 5, each column represents a possible conditional configuration. After deleting the conditions combination without cases, it can be observed that the consistency of all conditional configurations is more than 0.9, indicating that all cases meet the consistency condition (Ragin & Fiss, 2008). The overall consistency of the study is 0.9714, and the overall coverage is 0.5095, both of which are higher than the critical value. This suggests that the empirical analysis of the study was effective and had high explanatory power for necessity [38].

**Table 5:** Configuration analysis of Chinese port logistics.

Conditional variables	TE					~TE	
	H1a	H1b	H2	H3	H4	NH1	NH2
Infrastructure	●	●	●	⦻	⦻	⦻	●
Personnel	●	●	●	⦻	⦻	●	⦻
Structure	●		⦻		⦻	⦻	●
Hinterland Economy	●	●	●	●	⦻	⦻	⦻
Government		●	●	⦻	●	⦻	⦻
Openness	●	●	⦻	●	●	⦻	●
Informationization	●	●	●	●	⦻	⦻	⦻
Raw coverage	0.3566	0.2607	0.1739	0.1850	0.1648	0.3934	0.3363
Unique coverage	0.1064	0.0157	0.0308	0.0367	0.0315	0.0078	0.0180
Consistency	0.9979	0.9971	0.9872	0.9392	0.9244	0.8986	0.8807
Overall solution consistency	0.9714					0.8349	
Overall solution coverage	0.5095					0.4903	

Note(s): 1. Black circles (“●”) indicate the presence of a condition, and circles with a cross-out (““) indicate its absence.

Large circles indicate core conditions, small circles refer to peripheral conditions.

Blank spaces in a solution indicate a “don’t care” situation in which the causal condition may be either present or absent.

### 5.4 Configuration analysis

As shown in Table 5, there are four alternative schemes for improving operational efficiency in the Chinese port logistics industry. These schemes are mainly intermediate solutions combined with parsimonious solutions. The result shows that regions with good economic endowments have various paths to achieve high efficiency, while cities with poor economic endowments have only one path. Therefore, it is necessary to follow a specific configuration path, rather than imitate the path of other regions with good economic endowments. Meanwhile, the results of the fsQCA analysis further confirm the necessity of configuration analysis for port logistics. Accordingly, port cities with significant economic endowments have diverse paths to becoming highly efficient. In contrast, cities with insufficient economic endowments have only one path, further emphasizing the significance of this study. Port cities with poor economic endowments must follow a specific grouping path. They cannot blindly learn from the high-efficiency paths of cities with relatively robust economic endowments but need to create a personalized path according to their own conditions and endowments.

***Model 1*:** The configurations of Model 1 are H1a “Infrastructure * Personnel * Structure * Openness * Hinterland Economy * Informationization”, and H1b “Infrastructure * Personnel * Government * Openness * Hinterland Economy * Informationization”, this model ranks first with consistency at 0.997. The core conditions in Model 1 are Infrastructure, Personnel, Openness, and Informationization, which account for 35.6% and 26.1% of all highly efficient ports, respectively. In addition, port cities located in the Pearl River Delta and Yangtze River Delta comprise the core representative cases. The results show that the government support substitutes for industrial structures. Comparing H1a and H1b configurations, there is a link between industrial structure and government support. Their other condition variables are similar, suggesting government support is not necessary for port cities with better economic endowments, but it is important for backward ones. Moreover, a healthy industrial structure can achieve a high level of infrastructure, openness, and informatization, which can lead to a high level of efficiency in port logistics. Port cities that do not have a solid industrial structure should focus on improving the efficiency of port logistics through the implementation of policies and the allocation of funds.

***Model 2*:** Model 2 consists of only one configuration as H2 “Infrastructure* Personnel *~Structure* Government* Open* ~Hinterland Economy* ~Informatization”. This configuration requires a high level of infrastructure, personnel, government support, and openness at the same time. By comparing between configurations of H1 and H2, we found a substitution relationship between the industrial structure level of the city and the government’s support. For cities with good economic endowments, government support is not necessary. Port cities with good industrial structures can be adapted to high infrastructure, openness, and informatization, thus leading to a high level of efficiency. In contrast, port cities without forming a good industrial structure need strong support from the local government with policy guidance and capital tilt.

***Model 3*:** Configuration of model 3 is H3 “~Infrastructure *~Personnel *~Government * Openness *Hinterland Economy *Informationization”. The existence of high levels of hinterland economy and informatization are the core conditions, while infrastructure, personnel supply, and government support are the supporting conditions. By comparing with H2, the level of infrastructure, personnel supply, and government support are not decisively related to high efficiency. It can be explained that the support of the government can be reflected in the construction of infrastructure and the strength of attracting and attaining personnel. However, the diverse configuration results show that further avenues in the improvement scheme of technical efficiency of China’s port logistics should be conducted beyond the seven conditional variables in this paper. Considering the limitation of QCA method, it is difficult to reflect the progress of government support or the stage by adopting cross-sectional data.

***Model 4*:** For the configuration H4 in model 4, it is “~Infrastructure* ~Personnel* ~Structure* Government* Openness* ~Hinterland Economy*~*Informationization*”, which has the lowest consistency for 0.924. And this solution requires a high level of government support and openness. Its solution maintains 16.48% of all highly efficient samples. The main representative case of model 4 is Yantai (0.51, 0.95). Although Yantai is a significant transportation hub in the Bohai Sea Rim, the level of infrastructure, personnel, industrial structure, economic endowment, and informatization are relatively low in the national rankings and do not have comparative advantages. However, to optimize modern logistics development, Yantai has continuously improved the openness of the system. It has further improved the international trading and service capacity of bulk materials in recent years, building a port supply chain hub and industrial chain integration platform. In 2022, Yantai was approved as a port national logistics hub by China’s National Development and Reform Commission. Its technical efficiency has achieved a remarkable enhancement.

Additionally, two configurations of non-high efficiency are also included in this research, and the missing conditions occur with the absence of government support, the hinterland economy, and informationization. It can be found that the effective path to improve efficiency for port logistics in poor economic endowment is vigorously carried out policy support.

### 5.5 Robustness check

The fsQCA model can be tested for robustness by modifying the consistency threshold, the consistency of PRI, the number of condition variables, and the frequency of acceptable cases. Additionally, regarding the two criteria for measuring robustness of QCA results [38], such as the set-relation state of different configurations and the differences in fitting parameters. In this paper, the consistency threshold is increased from 0.8 to 0.85 and 0.9 by modifying the consistency threshold. The results show that the configurations are consistent with the results of the previous paper. In addition, consistency and coverage are consistent with the previous paper, which indicates that the results of this paper are robust.

## 6 Conclusion and discussion

Since the port logistics can support the development of the national economy, this research presents a guide to handling technical efficiency and improvement schemes for 19 active ports in China evaluated across 5 economic circles during 2011–2020. First, we adopt the DEA-BCC method to examine technical efficiency (CRSTE), pure technical efficiency (VRSTE), and scale efficiency (SCALE) efficiency based on the input-output system. In addition, this paper follows the study [20] conducting the fsQCA method to explain the schemes of efficiency improvement with a framework combined with both hard and soft environments, and the results are still valid after the robustness test.

Regarding the results of the DEA method, they can be listed as: (1) There are regional differences in efficiency performance distribution among economic circles. The results of some individual economic circles show inefficient and unreasonable input-output relationships. (2) The technical efficiency improvement in the Bohai Rim, Southeast Coast, and Pearl River Delta originates from scale efficiency. In contrast, the improvement in technical efficiency in the Southwest Coast and Yangtze River Delta economic circles mainly comes from pure technical efficiency. This indicates that the Bohai Rim, Southeast Coast, and Pearl River Delta economic circles can enhance their efficiency by expanding the scale of inputs while ignoring the improvement of management skills, and the scale of inputs grows too fast in logistics development while ignoring the effective configuration of management skills. (3) The heterogeneity on the southwest coast and the Yangtze River Delta indicates the importance of optimization of scale levels. It suggests that these economic circles fail to coordinate the relationship between pure technical efficiency and scale efficiency.

For the fsQCA results, we discovered 4 models with 5 configurations for improving technical efficiency for China’s port logistics. First, due to the absence of effective industrial agglomerations of the logistics industry in port cities, the development of port logistics requires strong support from the government with policy guidance and financial inclination, which contribute to the building of infrastructure and the introduction and retention of personnel. As a heavy asset industry, investment in infrastructure is an important social leading capital for port logistics. It can optimize the regional logistics industry structure and promote the technological efficiency of the logistics industry. Second, the port region should optimize the application of information technology in logistics construction and build a smart port. As the infrastructure of the port is upgraded, high-value-added functions will be expanded, digital construction will be strengthened, and the strength of the soft environment will be enhanced. Finally, government support is necessary for port logistics with a backward economic endowment. The government should establish a more open market system and expand the logistics industry market. Especially in underdeveloped port cities with backward economic endowments, the government should introduce private capital, enhance the degree of openness, and realize the diversification of property rights and market competition mechanisms. This will activate the logistics industry and thus, improve the efficiency performance of port logistics.

By presenting an assessment framework with a systematic methodology, the results provide guidelines for policymakers in China’s port logistics markets to make scientific arrangements to enhance performance in competitive economic environments. In addition, there are some limitations in this study. It is necessary to assess efficiency performance using various methods. The conditional variables for port logistics are not limited within this research since the lagging effect of the implementation of policies needs time to be confirmed. The cross-sectional data for 2020 has limitations in reflecting the progress of government support, which is also a limitation of the QCA method.

## Supporting information

S1 Data (XLSX)
